# Cellular Dynamics Drives the Emergence of Supracellular Structure in the Cyanobacterium, *Phormidium* sp. KS 

**DOI:** 10.3390/life4040819

**Published:** 2014-11-28

**Authors:** Naoki Sato, Yutaro Katsumata, Kaoru Sato, Naoyuki Tajima

**Affiliations:** 1 Department of Life Sciences, Graduate School of Arts and Sciences, University of Tokyo, Komaba 3-8-1, Meguro-ku, Tokyo 153-8902, Japan; E-Mails: yutaro21215@hotmail.com (Y.K.); naoT507@bio.c.u-tokyo.ac.jp (N.T.); 2 Department of Social Engineering, School of Engineering, Tokyo Institute of Technology, Ookayama 2-12-1, Meguro-ku, Tokyo 152-8550, Japan; E-Mail: sato.k.bf@m.titech.ac.jp

**Keywords:** cyanobacteria, *Phormidium*, supracellular structure, biofilm, spiral formation, emergence of order, slime gun model

## Abstract

Motile filamentous cyanobacteria, such as *Oscillatoria*, *Phormidium* and *Arthrospira*, are ubiquitous in terrestrial and aquatic environments*.* As noted by Nägeli in 1860, many of them form complex three-dimensional or two-dimensional structures, such as biofilm, weed-like thalli, bundles of filaments and spirals, which we call supracellular structures. In all of these structures, individual filaments incessantly move back and forth. The structures are, therefore, macroscopic, dynamic structures that are continuously changing their microscopic arrangement of filaments. In the present study, we analyzed quantitatively the movement of individual filaments of *Phormidium* sp. KS grown on agar plates. Junctional pores, which have been proposed to drive cell movement by mucilage/slime secretion, were found to align on both sides of each septum. The velocity of movement was highest just after the reversal of direction and, then, attenuated exponentially to a final value before the next reversal of direction. This kinetics is compatible with the “slime gun” model. A higher agar concentration restricts the movement more severely and, thus, resulted in more spiral formation. The spiral is a robust form compatible with non-homogeneous movements of different parts of a long filament. We propose a model of spiral formation based on the microscopic movement of filaments.

## 1. Introduction

Motile filamentous cyanobacteria, such as *Oscillatoria*, *Phormidium* and *Arthrospira*, are ubiquitous in terrestrial and aquatic environments. As early as in 1860, Carl Nägeli [1] described the motility and structural formation of these cyanobacteria in a pioneering publication, including various reports related to the motion of photosynthetic organisms, such as plants and algae. He described “*Hin- und Hergehen nebst Drehen um die Achse*”, “*Strahlen-förmiges Auseinanderweichen*” and “*Vereinigung in Bündel und Membranen*”. These correspond, respectively, to back-and-forth motion with rotation about the axis, radiating dispersion of filaments and collective formation of bundles and membranes. We are surprised to see that he already anticipated that particular types of motion of individual filaments were the origin of macroscopic structures formed by numerous filaments. Many filamentous cyanobacteria indeed form complex three-dimensional or two-dimensional structures, such as biofilm, weed-like thalli, bundles of filaments and spirals, depending on the media or supporting materials. Here, we call them collectively “supracellular structures”.

The formation of supracellular structures is distinct from the structures of common multicellular organisms in the following points: first, there is no well-defined structure specific to a species and environmental conditions. We can call them spirals or bundles, but the exact structure, as formulated by the number of constituent filaments and the size of the entire structure, is variable. Second, the structure is always changing with time or is always “under construction”. In other words, there is no pre-defined final form. Third, the macroscopic structure must have the flexibility that allows continuous movement of constituent filaments at the microscopic level. Finally, the form of the structure is a result of the interaction between the motile filaments and the environment and is not strictly governed by genetic programs, although the mechanisms of cellular motility and the production of extracellular substance must be genetically coded.

In the present study, we isolated a new strain of *Phormidium*, which shows the active formation of supracellular structures. After characterizing by 16S rDNA sequencing and electron microscopy, we analyzed, in detail, the movement of this cyanobacterium in single filaments and in spirals. We found that the agar concentration restricts the movement of filaments and, thus, promotes spiral formation. Based on all of the available data, we propose a dynamic model of spiral formation. 

## 2. Experimental Section

### 2.1. Strain and Culture

*Phormidium* sp. KS was originally isolated from a home aquarium and grown on BG-11 agar plates [2] under continuous illumination at 30–32 °C. Light was provided by fluorescent tubes at an energy flux of about 20 µE m^−2^ s^−1^. Since the cell filaments did not form colonies, but a network of filaments on an agar plate, the part of the agar containing newly propagated filaments was aseptically picked up and inoculated on to a new agar plate. Contaminating bacteria were removed by several rounds of growth on BG-11 agar plates containing 30 µg·mL^−1^ trimethoprim. Bacteria were further removed by growth in modified Bristol’s medium, which is normally used for the growth of green algae [3], but was found to be a good medium for this *Phormidium*. For liquid culture, the cells were inoculated in flasks containing BG-11 medium, and the flasks were placed under continuous illumination at 32 °C without shaking.

Short filaments (about 100 µm in length) suitable for the analysis of movement, were selected in the following way: a part of the cell filaments grown on a 0.5% agar plate was inoculated on a 1.5% agar plate. After 7 days, many filaments grew on the plate, and short filaments, probably produced as a result of the natural breakage of long filaments, were found at the periphery of the growing area. The movement of filaments on the plate was recorded as time-lapse video movies.

### 2.2. DNA Isolation and rDNA Sequencing

The filaments were collected into a microtube and disrupted by shaking with glass beads. Then, the nucleic acid fraction was obtained by extraction with phenol and chloroform, followed by ethanol precipitation. rDNA was amplified by PCR using commonly used primers (16S27F and 23S30R in [4]) and cloned by TA cloning. Sequencing was performed by conventional Sanger sequencing using various primers listed in [4]. The rDNA sequence was registered in the DDBJ database under Accession Number AB510147. 

Large-scale preparation of genomic DNA using ultracentrifugation over a CsCl gradient was performed essentially as described in [5]. Illumina MySeq sequencing of the genomic DNA was performed by Takara Bio Inc. (Otsu, Japan). Sequences (paired-end reads of 150 bases long) were assembled by Velvet software [6] using a Linux workstation. The nucleotide sequences of the three *hps* gene clusters and the *hmp* gene cluster were deposited in the DDBJ database under Accession Numbers AB992254, AB992255, AB992256 and AB992257 (*hmp*), respectively.

For phylogenetic analysis, rDNA sequences of related organisms were downloaded from the Ribosomal Database Project release 10 [7]. Sequence alignment was prepared by the Clustal X program [8], and initial phylogenetic analysis was performed by the Phylip package version 3.67 [9]. After removing redundant sequences, a neighbor joining tree was constructed by the MEGA software version 4 [10]. The options were: pairwise deletion, maximum composite likelihood model, substitutions, including transitions and transversions, homogeneous among lineages, uniform rates and 1000 bootstraps. The maximum likelihood tree was constructed by the TreeFinder software, version of March 2008 [11]. The J1 + G model was used according to the result of the ModelProposer command in TreeFinder.

### 2.3. Transmission Electron Microscopy

A part of the agar plate bearing *Phormidium* filaments was cut out with forceps and fixed with 2.5% glutaraldehyde in 0.1 M sodium cacodylate buffer (pH 7.4) for 2 h and, then, postfixed overnight with 1% osmium tetroxide. The samples were dehydrated in increasing ethanol series and propylene oxide and, finally, were embedded in Epon resin (Nisshin EM, Tokyo, Japan). Ultrathin sections were stained with uranyl acetate and lead citrate. Images were obtained by an electron microscope, model 1200EX (JEOL), operated at 80 kV.

### 2.4. Recording and Analysis of Movements

The macroscopic view of cell growth on the plates was observed with a low-magnification microscope, model MZ16FA (Leica Microsystems, Tokyo, Japan), and images were recorded with a CCD camera, model VB-7010 (Keyence, Osaka, Japan). The movement of filaments was observed by a microscope, model BX-60 (Olympus, Tokyo, Japan), with a CCD camera, model DP70 (Olympus). For recording rapid motions, another CCD camera, Rolera-XR (QImaging, Surrey, BC, Canada), with StreamPix software (version 3.45.0, NorPix Inc., Montreal, QC, Canada), was used. Image processing and analysis were performed with ImageJ software (version 1.41 for Mac OS X [12]).

## 3. Results and Discussion

### 3.1. Characterization of Phormidium sp. KS 

**Figure 1:**
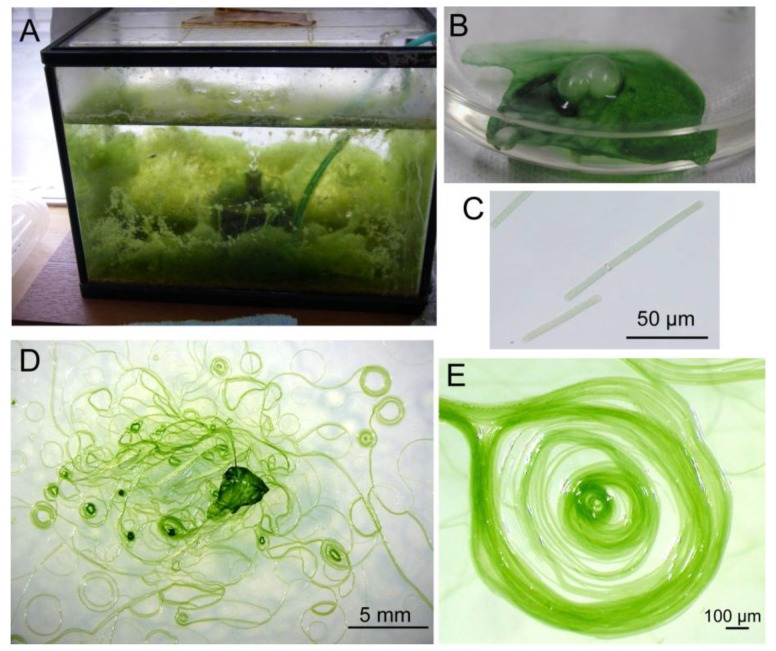
Different views of growing *Phormidium* sp. KS. (**A**) *Phormidium* is growing like seaweed in an aquarium; (**B**) Biofilm of *Phormidium* with an air bubble formed spontaneously; (**C**) Individual short filaments of *Phormidium*; (**D**) Spirals formed on an agar plate; (**E**) Enlarged view of a spiral consisting of many filaments.

**Figure 2:**
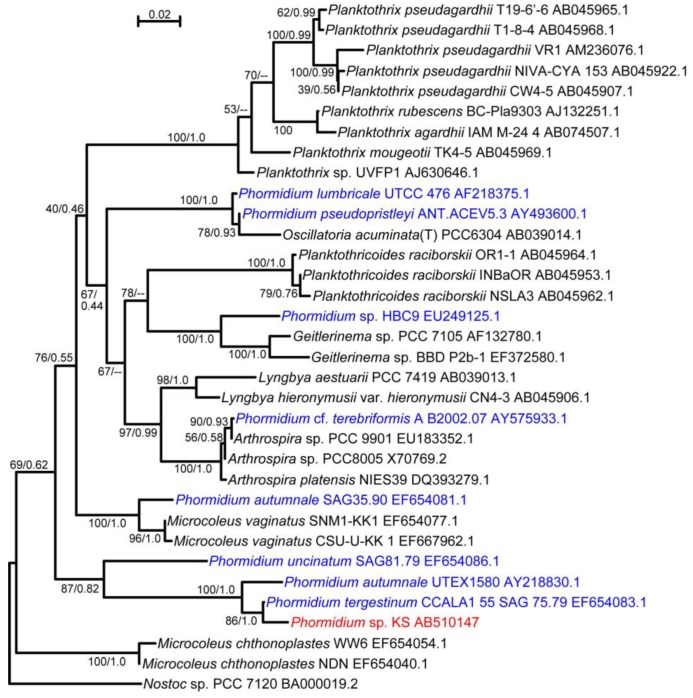
Maximum likelihood (ML) tree of *Phormidium* sp. KS and related cyanobacteria based on 16S rDNA sequences. Each set of values separated by a slash on each branch indicates the confidence values with ML and neighbor joining (NJ) methods. Hyphens indicate that the branch was not supported by the NJ method. Various strains with the name *Phormidium* are in blue, whereas the strain KS is shown in red. The accession number in GenBank is shown with the strain name. *Nostoc* sp. PCC 7120 was added as an outgroup. The uppermost bar indicates the scale for the branch length (substitutions per site).

We obtained a filamentous cyanobacterium from an aquarium (Figure 1A) and purified it by repeated plating. Since the filaments did not form colonies, a newly growing tip of a filament on a virgin agar plate was picked up and inoculated on a new plate. Sequencing analysis of the rDNA suggested that this cyanobacterium was a member of the *Phormidium* clade (Figure 2). It has been shown that various cyanobacterial strains under the name *Phormidium* are, in fact, paraphyletic [13]. In this respect, our isolate was included in clade number 11 [13]. The closest relative was *Phormidium tergestinum* CCALA1 55 SAG 75.79. We call our isolate *Phormidium* sp. KS.

### 3.2. Divergent Structures Formed by Phormidium

Figure 1 shows various structures formed by *Phormidium* sp. KS. In the aquarium with a continuous flow of water, it formed “an algal forest”, as seen in Figure 1A. The filaments grew according to the stream of water, and the growth continued over the entire space of the aquarium. In a static liquid culture (Figure 1B), a mat consisting of reticulate filaments was formed at the surface, and occasionally, a part of it swelled to form a visible bubble or balloon. A mat became a large membranous structure, if enough space were available.

**Figure 3:**
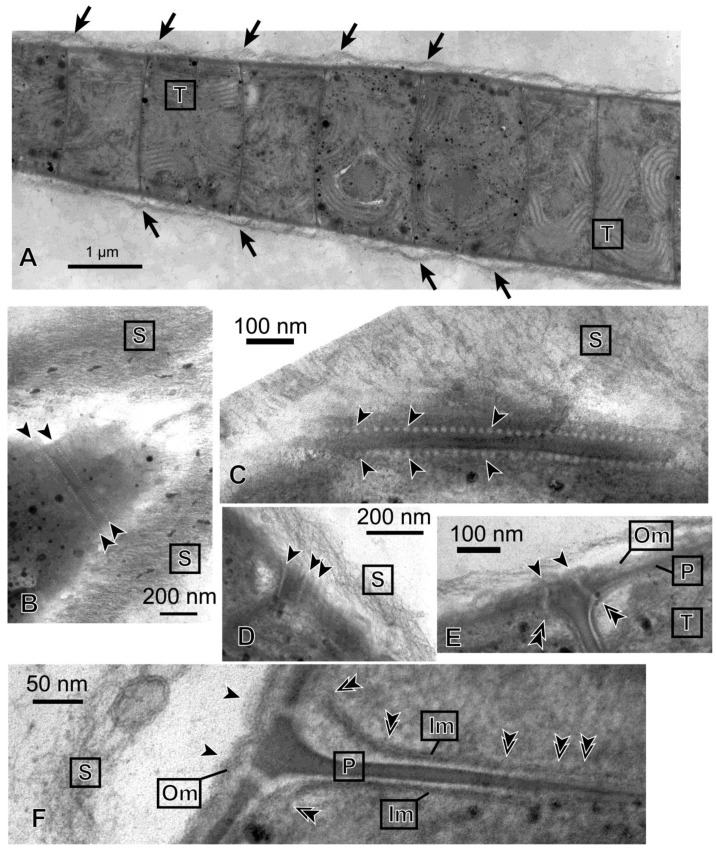
Transmission electron micrographs of *Phormidium* sp. KS. (**A**) Longitudinal section of a part of a long filament. Arrows indicate that the sheath layer is separated from the cell body at each septum. This large panel is a collage of three different images, which were assembled with imaging software Photoshop (Adobe); (**B**) The tangential section of a tapered tip of a filament. A row of junctional pores on each side of the septum is indicated between the arrowheads; (**C**) Close-up view of the rows of junctional pores (arrowheads); (**D**) Longitudinal view of junctional pores (arrowheads) seen in an obliquely cut section; (**E**) Junctional pores spanning the entire cell envelope. Each arrowhead indicates the site of connection of the junctional pore and outer membrane. Each double arrowhead indicates the site of connection of the junctional pore and inner membrane; (**F**) Enlarged view of a section of a septum. Two junctional pores spanning the left surface of the cells are shown, each between an arrowhead and a double arrowhead. The bulges of membrane identified in the inner membrane facing the septum (double arrowheads) could be the sites corresponding to the location of junctional pores (we assume that the pore-forming tube is oriented nearly vertically to the image). Note that the inner membranes were obscured at their left end, because the membranes were not cut at a right angle at these sites. Im, inner membrane; Om, outer membrane; P, peptidoglycan layer; S, sheath; T, thylakoid membrane.

Fragmented filaments that formed spontaneously grew as individual short filaments (Figure 1C). Electron microscopy showed that each filament consisted of many disk-shaped cells stacked closely (Figure 3A). On agar plates, the filaments continuously moved back and forth with a period of 5−10 min. In addition, the filament rotated clockwise around its long axis during the forward movement (Supplemental Movie S1). In this movie, the tip of the filament showed a precession movement during the forward movement, but this was not always observed clearly in longer filaments. Apparently, the filaments moved within a transparent sheath, which itself consisted of many thin fibers, as seen by electron microscopy (Figure 3B,C, Label “S”). When a small plug of agar medium containing cell filaments was placed on a new agar plate, filaments grew out from the plug. Many filaments formed spirals after a week or two (Figure 1D). Each spiral is formed with a single filament (or a few filaments) that turned around many times to form a bundle of filaments arranged in the shape of a ring. Figure 1E shows a complex spiral formed by several filaments after a long culture period. The number of filaments that formed a bundle was variable. Such thick bundles were visible by the naked eye without magnifier. 

In *Pseudanabaena* [14] or *Limnothrix* [15], which form filaments consisting of trains of long cylindrical cells, the filaments move as bundles and form reticulate mesh-like structures. However, no spiral is formed. The formation of the supracellular structure is certainly related to cell motility, but the formation of various types of structures may be dependent on cyanobacterial species.

### 3.3. Ultrastructure of Phormidium Filaments

Figure 3 shows the ultrastructure of a filament of *Phormidium* sp. KS. The entire filament was very long, and Figure 3A shows only a part of it. The individual cells were thin discs that were stacked to form a filament. Within a cell, parallel rows of thylakoids (labeled “T”) were seen. The entire filament was covered with a thick sheath, which consisted of thin fibrous materials (labeled “S”). The sheath was separated from the cell body at the sites of septa. This is compatible with the idea that mucilage (or slime) is secreted from the junctional pores [16], which were found to align on both sides of a septum (Figure 3B,C). The size of a pore was about 10−15 nm in diameter, and the interval was about 20 nm. The pores seemed to penetrate the outer membrane, peptidoglycan layer and inner membrane like straight tubes (Figure 3E,F). The diameter of the tube seemed larger, however, when it passed the space between the peptidoglycan layer and inner membrane. In an obliquely cut section (Figure 3D), several pores in alignment were found to penetrate the cell envelope. The axis of the pore-forming tubes seemed to incline with respect to the axis of the filament, so that the excreted mucilage could generate a helical flow. In the interior region of an inner membrane facing a septum (Figure 3F), bulge-like deformations were found to locate at an interval of 20 nm, suggesting that these correspond to the location of junctional pores. 

### 3.4. Growth of Filaments on Agar Plates

**Figure 4:**
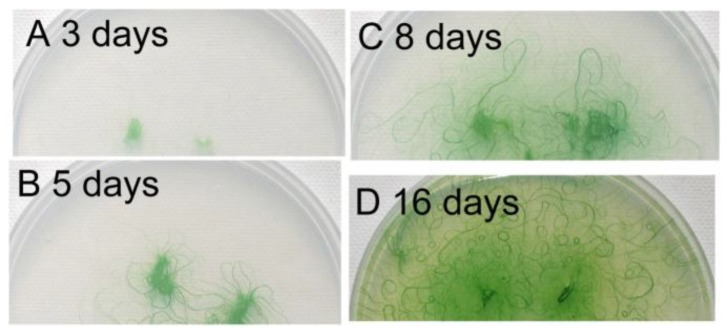
Growth of filaments on an agar plate. Small agar plugs containing filaments of *Phormidium* were placed on a 1.5% agar plate (diameter: 9 cm), and the cells were allowed to grow under continuous light at 30 °C. An identical plate was photographed at different time points. (**A**) Day 3; (**B**) Day 5; (**C**) Day 8; (**D**) Day 16. Note that many small spirals are formed after 16 days.

Figure 4 shows the growth of filaments on an agar plate, beginning from small agar plugs. The filaments developed radially during the first five days, but then, the direction of growth was diversified, because of the crowding of the filaments. At the same time, bundles of filaments were formed and became clearly visible (Figure 4C). We can see some U-shaped bundles of filaments. Finally, many small circles became visible (Figure 4D). These results indicate that the formation of spirals was an outcome of the dynamic growth of filaments accompanying the movement of filaments. Although any single short filament was straight (Figure 1C), the curved shape of filaments developed with time, with the elongation of filaments by cell division.

**Figure 5:**
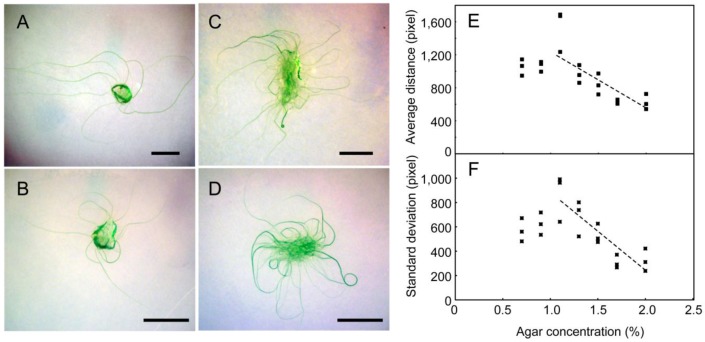
The effects of agar concentration on the growth development of filaments: (**A**) 0.7%; (**B**) 1.1%; (**C**) 1.5%; (**D**) 2.0%; Bars: 1 cm. (**E**) Statistics of the average spread of filaments; (**F**) Standard deviation of the spread of filaments. This is a statistics of the distances of all pixels on the filaments from the starting point. Note that filaments grew in a radial manner at low concentrations of agar, whereas at higher concentrations, both the average distance and standard deviation were reduced. The decrease in standard deviation was an indication of spiral formation.

We then analyzed the effects of agar concentration on growth development (Figure 5). Below 1.1% of agar, the filaments became submerged within the agar (Figure 5A), and thus, the propagation of filaments was attenuated. Note that under these conditions, no spirals were formed, even after a long time. The growth was fastest on 1.1% agar. Above this concentration, the propagation decreased with increasing agar concentration (Figure 5E). Since the growth of filaments was restricted on the surface of the plates above 1.1% agar, this decrease in development was a result of the stickiness of filaments to the agar surface, rather than a decrease in growth itself. As judged from the increase in green area (or precisely, the green channel intensity in pixels) in the images, the growth of cells was not markedly affected. Apparently, most of the filaments gradually curved counterclockwise on the plate under these free-growing conditions without crowding (Figure 5C,D). This is likely to reflect the clockwise rotation of each filament around its axis during the movement, as explained later (see Figure 9B). As shown in Figure 5F, the standard deviation of filament growth also decreased with agar concentration, which corresponded to the local assembly and organization of filaments (including spiral formation) at the periphery of the growth area.

### 3.5. Analysis of Movement of Filaments

**Figure 6:**
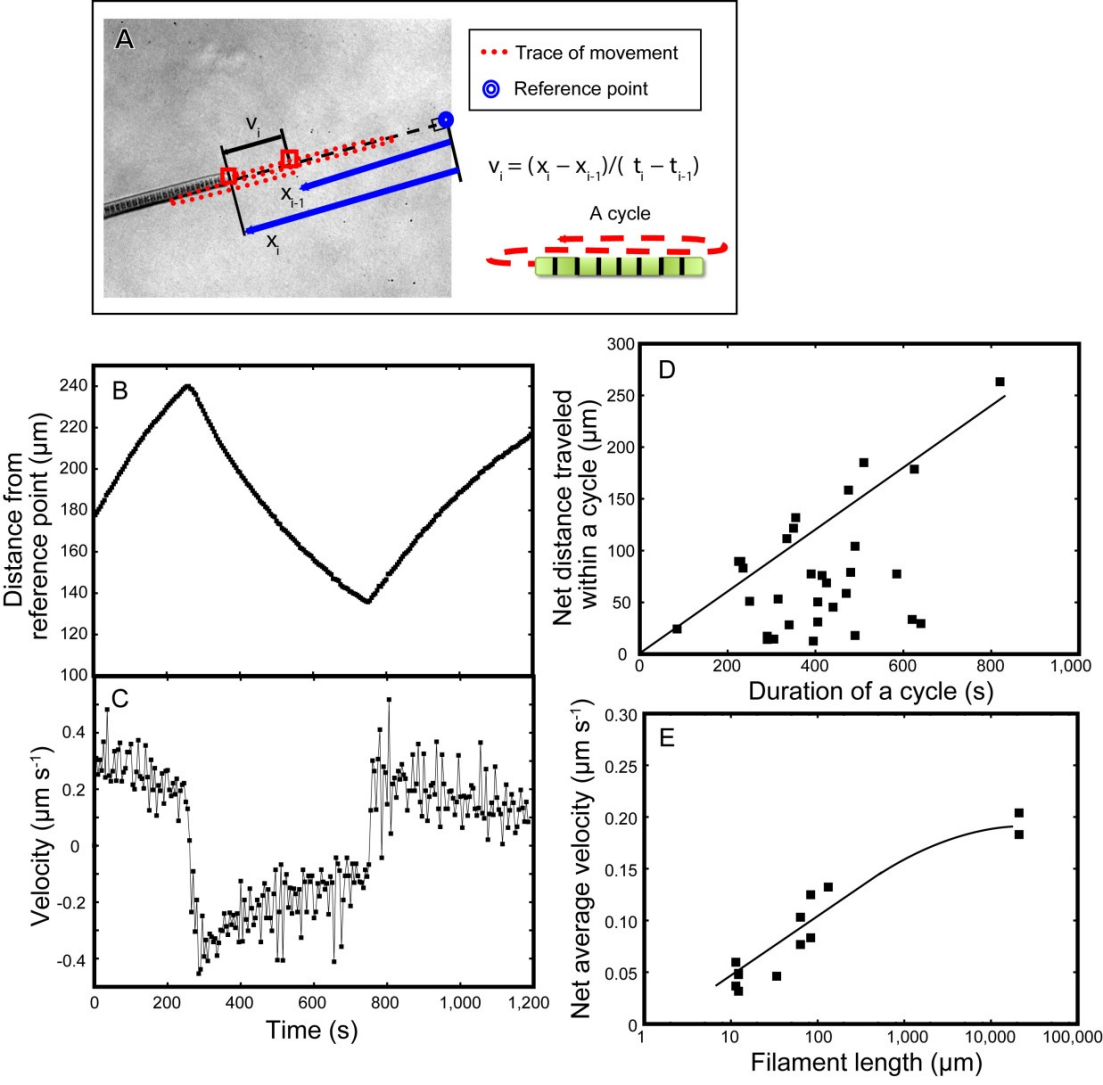
Mathematical analysis of the movement of filaments on 1.5% agar plates. (**A**) Measurement of the displacement of a filament and calculation of the velocity; (**B**) Displacement of a tip of filament with respect to a fixed reference point; (**C**) Velocity of a tip of filament; (**D**) Relationship between the duration and length of the displacement of an oscillatory cycle. The line indicates the upper limit of displacement; (**E**) The relationship between filament length (in logarithmic scale) and average velocity. The line indicates that the net velocity increased to attain an upper limit asymptotically.

We then analyzed the movement of individual short filaments (Figure 6). A single filament moved back and forth (Figure 6B). Here, the position *x_i_* of a tip of filament with respect to a reference point was traced with time *t_i_* (Figure 6A). The velocity *v_i_* was calculated and plotted against time *t_i_* (Figure 6C). Curiously, the velocity was maximal immediately after each reversal of direction. The decline in velocity was exponential. 

Such exponential decline in velocity might not be compatible with various mechanisms of movement using motor proteins, such as those with pili [17] or flagella (not present in *Phormidium*), because such mechanical motors should act at a constant rate. We found no description of an exponential or time-dependent decline in velocity in published papers on cellular movement by Type IV pili or focal adhesion. However, the mechanism involving mucilage secretion from junctional pores [16] might be compatible with the observed time course in velocity, if we suppose that the secretion is maximal at the initial burst and then decreases with time. 

As the movement reverted repeatedly, the overall average velocity of net movement was lower than the maximum velocity (Figure 6D). Only in some exceptional cases (the straight line in Figure 6D), net displacement with maximum velocity was achieved. In other cases (the points below the line), the average velocity was quite limited at about 0.1−0.15 µm·s^−1^. Long filaments tend to achieve large net displacement (Figure 6E), maybe because the forward and backward movements became asymmetric with filament length. However, we still do not know exactly the reason for this asymmetry.

**Figure 7:**
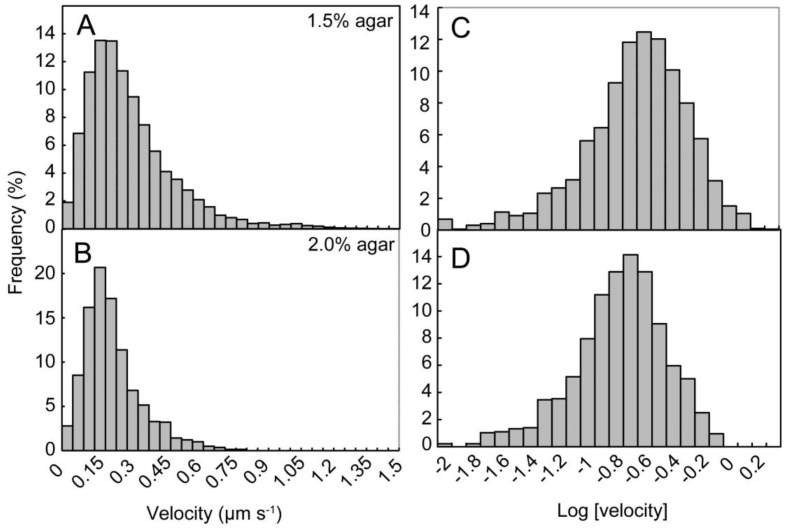
The effects of agar concentration on the distribution of velocity. (**A**) Distribution of velocity on 1.5% agar plates (*n* = 8882); (**B**) Distribution of velocity on 2.0% agar plates (*n* = 1397); (**C**) Distribution of the logarithm of velocity on 1.5% agar plates; (**D**) Distribution of the logarithm of velocity on 2.0% agar plates. Here, each velocity value was taken as a positive value. Note that the distribution of velocity is similar to the log-normal distribution, and the average velocity was lower on 2.0% agar plates.

Figure 7 shows the effects of agar concentration on the velocity of filaments over all time points during the measurements. Average velocity was 0.216 and 0.162 µm·s^−1^ on 1.5% and 2.0% agar plates, respectively. This difference was significant at the 5% level. The distribution was similar to a log-normal distribution (Figure 7C,D), rather than a normal distribution. In fact, such distributions are alternative representations of Figure 6C, showing exponential decline in velocity. From these results, we can estimate the terminal velocity, which was 0.169 and 0.148 µm·s^−1^ on 1.5% and 2.0% agar plates, respectively. The time constant of decline was 99 and 108 s^−1^, respectively. 

### 3.6. Conservation of *Hps* and *Hmp* Genes in Phormidium 

**Figure 8:**
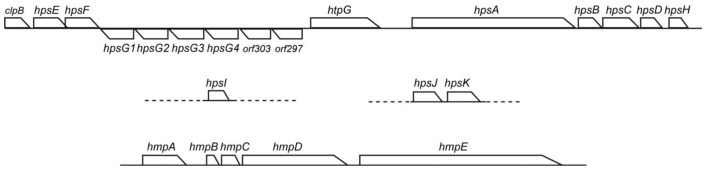
The *hps* and *hmp* gene clusters in *Phormidium* sp. KS. The large *hps* gene cluster is contiguous, whereas *hpsI* and *hpsJ/K* are located on different contigs, respectively. The *hmp* gene cluster is located on a further different contig.

A motility system with polysaccharide secretion was recently characterized in hormogonia of *Nostoc punctiforme* [18]. The *hps* locus includes genes encoding pseudopilins (HpsB, C, D and H) and glycosyltransferases (HpsE, F, G, I and K), as well as functionally unknown proteins (HpsA and J). This system is conserved in filamentous cyanobacteria and is a plausible candidate for the mechanism of motility with mucilage secretion from junctional pores [19]. We indeed detected *hps* genes in *Phormidium* sp. KS (Figure 8). The E-values of *hpsA*,* B*,* C*,* D*,* E*,* F*,* H*,* I*,* J* and* K* for the *Nostoc* ortholog were 2e−26, 2e−8, e−15, e−14, e−130, e−112, 2e−29, 3e−63, 4e−49 and e−143, respectively. There was another set of *hpsE* and *F* at a distant position within the genome. There were four copies of *hpsG* arranged in tandem. The E-values of these four copies with respect to the *Nostoc* *hpsG* was from 2e−94 to 3e−81. It should be noted that apparently unrelated genes, *clpB*, *htpG*, *orf303* and *orf297*, were also present in the large *hps* cluster. The *orf303* encodes a highly conserved sulfotransferase, whereas the *orf297* encodes a family 2 glycosyltransferase. In addition, the *hmp* genes [19,20], which are involved in the regulation of *hps* genes, were also conserved in the genome of *Phormidium* sp. KS (Figure 8). The E-values of *hmpA*,* B*,* C*,* D* and *E* to the *Nostoc* ortholog were e−130, 7e−58, 4e−69, 0.0 and 0.0, respectively.

### 3.7. Model of Filament Movement and Spiral Formation 

**Figure 9:**
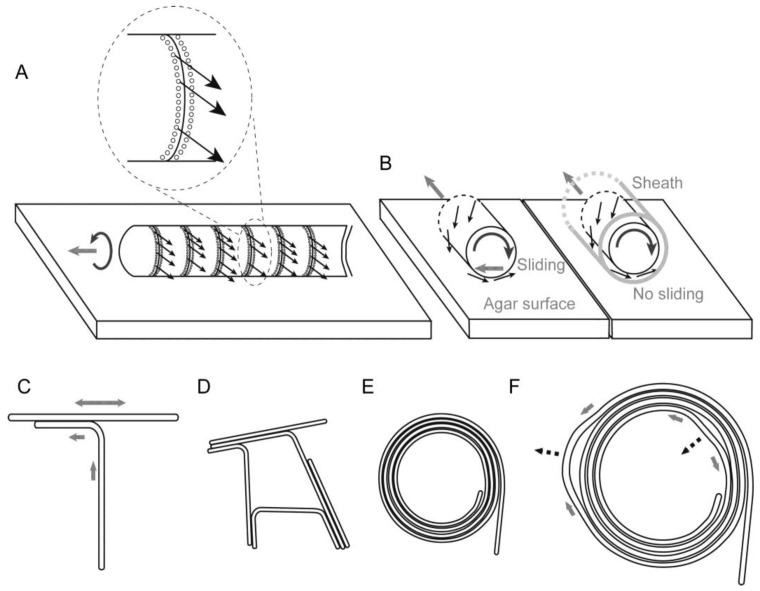
Helical flow-driven rotation model explaining the formation and stability of the spiral structure. Each thin arrow indicates a hypothetical flow of mucilage. Mucilage might be secreted from all of the junctional pores (shown by tiny circles aligned in a row adjacent to a septum) in the lagging pole of each cell, but only a few arrows are drawn for clarity. A grey thick arrow indicates the direction of filament displacement. This figure depicts only a phase of left-hand movement during the continuous back and forth movement. (**A**) Movement of a filament by excretion of mucilage. The inset is an enlargement of a septum; (**B**) Rotation of a filament by helical flow of mucilage. (**Left**) Movement on an agar substratum without a previously formed sheath, (**right**) movement within a sheath. Lateral sliding is provoked in the left case; (**C**) The encounter of two filaments directs a left-hand turn; (**D**) A meshwork structure formed by many filaments stuck together; (**E**) A spiral formed by a filament. The counterclockwise curvature of filament on solid agar eventually engenders a spiral; (**F**) Robustness of a spiral structure against local non-uniform movement. The movement of a filament is not identical throughout a single filament. A spiral structure can accommodate such non-uniform local movements shown by grey thick arrows and deforms, as shown by the broken line arrows.

Based on these results, we describe the motion of *Phormidium* filaments in the following way: a short filament moves by excreting mucilage or slime (uncharacterized polysaccharides) from the junctional pores aligned in a single row located at the lagging end of each cell. The junctional pores that seemed to be inclined with respect to the axis of the filament (Figure 3D) generate a helical flow of mucilage surrounding the filament (Figure 9A). It has been suggested that the helical flow of mucilage was directed by the rows of oscillin fibrils [21], which were visualized by a cryoEM technique in *P. uncinatum*. Unfortunately, our current electron microscopic procedure poorly preserves the surface structures, such as the S-layer and oscillin fibrils, which were not visible in Figure 3. We are not sure if inclined junctional pores (this is still a hypothesis, but they look inclined in Figure 3D) alone can produce a helical flow of mucilage sufficient for driving the rotation and movement of a filament. The flow-aligning role of fibril arrays documented 40 years ago is still an attractive hypothesis [22]. It is not clear yet, however, if oscillin is present in *Phormidium* sp. KS, because no homolog with high similarity (at most 34% identity) was found to be encoded in the genome. Oscillin was discovered in a related strain, *P. uncinatum* (see the phylogenetic tree in Figure 2), and is a very glycine-rich large protein having a calcium-binding activity. Even though an ortholog is not present in the strain KS, another protein with similar features could function to cause the helical flow of mucilage. 

The velocity of filament displacement is likely to be determined by the force of secretion and the viscosity of the mucilage, the latter of which is dependent on dehydration by surrounding agar. According to the “slime gun model” originally proposed in *Myxobacteria* ([17] adapted from [23]), hydration of the polysaccharide in the nozzle chamber of the junctional pore complex causes the filling of the nozzle chamber, which eventually generates a counter-force of the nozzle walls that results in the ejection of the slime. This is consistent with our current findings in *Phormidium*: first, the velocity of movement was maximal just after the reversal and then declined exponentially. This is hardly expected to occur if other kinds of mechanical devices (Type IV pili or focal adhesions [17]) drive the movement; second, a higher concentration of agar substratum decreased the velocity. This decrease could be explained by a lower rate of hydration of polysaccharide within the nozzle. This model also explains periodic changes in movement: once the content is ejected, a certain time is needed to refill the nozzle with hydrated slime. The *hps* gene clusters in *Phormidium* sp. KS encode eight glycosyltransferases and four pseudopilins [18] (see Figure 8), which are, respectively, involved in the synthesis and secretion of polysaccharides. The pseudopilins are known as components of the secretion/motility machineries [24]. The secretion of slime might be regulated by a mechanism similar to the formation of Type IV pili, by the secretion of a finite amount of pilin proteins. Nevertheless, the mechanism of how a single row of junctional pores is selected and (probably) synchronized remains unknown. The reversal of the direction of movement is likely to be caused by switching the active row of junctional pores, as suggested in [16], and a recent model suggested that this might be mediated by the *hmp* chemotaxis-related genes (Figure 8) [18,19,20]. Another possibility could be a change in membrane potential, but we still need studies on this point. 

It is also to be noted that the movement is not uniform along the entire filament, because larger net displacement was found in longer filaments (Figure 6E). This is clearly seen in Supplementary Movie S2. In this respect, the synchronization of movement is a local phenomenon within a single filament.

The spiral formation of *Phormidium* filaments on the agar surface (more than 1.1%) can be explained in the following way: during the course of growth, each filament curved counterclockwise (Figure 5), presumably due to its inherent rotatory motion around its long axis accompanying the forward movement (Figure 9A,B). This should be considered carefully, because a forward movement and a backward movement might not be on the same track, if the trail should be curved. This is obviously not the case (Supplementary Movie S1). Once formed, a sheath guides the movement of a filament. That is why repeated movements within a sheath do not normally change the trail. If we consider a forward movement on a virgin substratum, then the helical excretion of mucilage is expected to invoke a reaction that laterally slides the filament to the left (Figure 9B, left). In the next backward movement, the filament would follow the trail without lateral sliding (Figure 9B, right). As cells divide, the filament becomes longer. A longer filament will intrude into a new area of agar substratum. In this way, the intrusion of a growing filament into a virgin agar surface would curve the filament counterclockwise (Figure 5). In addition, even a short, single filament is slightly twisted and showed precession movement, as seen in Supplementary Movie S1. This would add an orienting role to the movement of a filament.

The tendency of counterclockwise curvature should be enhanced by an encounter with another filament running across ahead of an advancing filament (Figure 9C). Such an encounter will lead to either a meshwork of filaments (Figure 9D) or a spiral (Figure 9E), although we still do not know exactly the process of spiral formation. However, we can easily understand the robustness of a spiral shape (Figure 9F). The movement of a filament or filaments forming a spiral is not uniform. Detailed observation (Supplementary Movie S2) showed that some part moves clockwise, while another part goes counterclockwise, and such non-synchronous movements changed with time. By this non-uniform motion, one part is relaxed, while another part is tightened (Figure 9F). Nevertheless, the overall shape of a spiral is tolerant to this non-uniform motion by changing the local radius of curvature. Such robustness of form explains that a spiral persists once it is formed.

## 4. Conclusions 

In this article, we presented macroscopic and microscopic analysis of the motion of filaments in *Phormidium* sp. KS, as well as ultrastructural evidence for the presence of junctional pores probably excreting mucilage. The kinetic characteristics of the movement can be explained by the mucilage excretion or “slime gun” mechanism, because the velocity of movement was maximal just after the reversal of direction and declined exponentially. The rotation around the filament axis accompanying the movement can be understood if the junctional pores, slightly inclined with respect to the filament axis, generate a helical flow of mucilage over the surface of the filament. Helically-arranged fibrils [22] that were not seen in our electron microscopic images could also contribute to helical flow of mucilage. Based on these, we propose the following scenario for the formation of spirals of filaments:

(1) If the agar medium is solid enough to prevent the submergence of filaments, the growth and movement of the filaments are restricted to the surface of the agar substratum.
(2) Each filament becomes curved during its growth, because its rotation around its axis accompanying its forward movement tends to cause left-hand sliding of the filament on a virgin substratum.
(3) Upon encountering another filament, a filament turns to the left, due to its inherent rotation around its axis. This motion occasionally initiates a spiral. After a spiral is formed by a single filament, the bundle of filaments continuously thickens, due to cell growth.
(4) Once a spiral is formed, it is maintained as a robust structure, which is tolerant to the non-uniform movement of different parts of a constantly growing filament.

The formation of a biofilm is still a more complex process. All filaments within a biofilm are always moving back and forth. The meshwork (Figure 9D) might be another type of robust structure that tolerates the non-uniform motion of different parts of filaments and is a basic structure of a biofilm.

Supracellular structure is an interesting field of research, which not only gives important insights into basic studies, but also provides clues to applied sciences. For example, costless cell recovery using biofilm is a promising technology in biofuel production. In addition, the biofilm mat of *Phormidium* can be used as a natural polymer. The supracellular structure is also interesting from the viewpoint of evolution. The formation of a two- or three-dimensional structure of bacteria without a genetically determined program of construction could be an alternative way of the biological evolution of complex structures that are possible within the limitation of the bacterial genome and cell structure or, in short, within the limit of prokaryotes. 

In a more general sense, supracellular structure also provides an interesting model of the emergence of order from unordered motion. Microscopic movement of cells or filaments is necessary for the formation of macroscopic order or structures, but there is no predefined program to direct the construction of a spiral with a definite size at a definite time. The machinery of mucilage excretion is obviously encoded by the genome, but the genome does not provide an instruction for how to construct a spiral. In this sense, the formation of this supracellular structure is not said to be genetically programmed. This statement does not mean that none of the supracellular structures are genetically determined. We point out, however, that some non-genetically determined processes are participating (often as a major cause) in various supracellular structures. This is an interesting feature of supracellular formation that we will have to study in more detail in the future. We will also have to mention that eukaryotes might also use a similar strategy for structure formation, at least in part of a complex, genetically programmed pathway of development.

## Supplementary Materials

Supplementary materials can be accessed at: http://www.mdpi.com/2075-1729/4/4/819/S1.

Movie S1: Movement of a single filament. This movie was originally recorded at one frame every 5 s. The file in avi format is set to view at a frame rate of 2 frames per second.

Movie S2: Movement of a filament within a spiral. The same frame rate was used as above. Note that the movement was not uniform.
